# *In vitro* assessment of antibacterial activity from *Lactobacillus* spp. *strains* against virulent *Salmonella* species isolated from slaughter animals in Benin

**DOI:** 10.14202/vetworld.2019.1951-1958

**Published:** 2019-12-13

**Authors:** Alidehou Jerrold Agbankpe, Tamegnon Victorien Dougnon, Roubaya Balarabe, Esther Deguenon, Lamine Baba-Moussa

**Affiliations:** 1 Research Unit in Applied Microbiology and Pharmacology of Natural Substances, Research Laboratory in Applied Biology, Polytechnic School of Abomey-Calavi, University of Abomey-Calavi, Cotonou, Benin; 2 Laboratory of Biology and Molecular Typing in Microbiology, Faculty of Science and Technology, University of Abomey-Calavi, Cotonou, Benin

**Keywords:** antibacterial activity, Benin, *Lactobacillus* spp, probiotic, *Salmonella* spp

## Abstract

**Background and Aim:**

*Salmonella* spp. are among the world’s leading foodborne pathogens, found naturally in the intestines of many animals. Lactic acid bacteria, mainly *Lactobacillus*, are a promising alternative to antibiotics for animal and human health. This study aimed to assess the *in vitro* antibacterial activity of *Lactobacillus* spp. strains against virulent *Salmonella* spp. isolated from slaughter animals in Benin.

**Materials and Methods:**

Eleven samples of raw cow’s milk, five samples of breast milk, and six infant stool samples were taken. From these samples, strains of *Lactobacillus* were isolated and identified. The probiotic potential of each of the identified strains was characterized, and finally *in vitro* antibacterial activity of these strains was evaluated against three virulent strains of *Salmonella* spp. and a reference strain of *Salmonella* Typhimurium ATCC 14028.

**Results:**

Out of the 22 samples collected, 20 strains of *Lactobacillus* spp. were isolated and identified. These strains included *Lactobacillus plantarum* (30%), *Lactobacillus delbrueckii* (25%), *Lactobacillus casei* (25%), *Lactobacillus salivarius* (15%), and *Lactobacillus acidophilus* (05%). Characterization of the probiotic potential of these strains showed that only 16 strains were resistant to pH=1.5. Fourteen of them were able to withstand the simulated gastric juice (pH 1.5+pepsin). The 14 probiotic strains showed very good antibacterial activity against virulent strains of *Salmonella* spp. with inhibition zone diameters ranging from 12.36±0.03 mm to 35.33±0.05 mm (R values>6 mm).

**Conclusion:**

From this study, *Lactobacillus* strains isolated from raw cow milk, breast milk, and infantile stool might be used as some valid candidates for probiotics. It also represents good alternatives for antibiotics in the fight against animal and human salmonellosis.

## Introduction

Salmonellosis, an infection caused by the bacteria called *Salmonella*, is one of the leading causes of foodborne illness in humans and animals. Although animals, such as pigs, chickens, and cattle, infected with *Salmonella* do not normally show symptoms, carcasses and meats are the main reservoirs of the pathogen [1]. In the United States and the European Union, outbreaks of salmonellosis have been reported each year. In the world and humans, there are approximately 93.8 million cases of salmonellosis causing approximately 155,000 deaths/year [2,3]. Minor salmonellosis (non-typhoid) causes 3.4 million infections and 681,000 deaths worldwide. In Africa, non-typhoid *Salmonella* is a major cause of bacteremia, particularly in children and people with compromised immune functions [3]. Antibiotics are used for the prevention and treatment of infectious diseases. However, the appearance of antibiotic-resistant bacteria in humans and animals is linked to the widespread use of antibiotics in farmed animals [4].

The major public health problem caused by non-typhoid *Salmonella* was amplified by the emergence of multidrug-resistant strains [5]. It has been recognized that the emergence of antimicrobial resistance, particularly multidrug resistance to ampicillin, chloramphenicol, and cotrimoxazole, has complicated the treatment and management of salmonellosis [6]. Increased antibiotic usage is a key factor in the emergence of antibiotic-resistant pathogens. Thus, there is an urgent need to develop alternatives to antibiotics. The use of probiotics is then given great attention as an alternative to antibiotics [1]. Probiotics are widely studied and implemented in several applications such as the prevention of food poisoning or the treatment of certain gastrointestinal disorders [7]. Identified as an alternative treatment for *Salmonella* infections, probiotics are associated with fewer side effects and better safety. Several studies indicate that different *Lactobacillus* strains could inhibit the adhesion of *Salmonella*, thus reducing colonization and preventing infection [7,8].

In the search for an alternative solution to the use of antibiotics in the treatment of Salmonellosis in Benin, the present study was initiated. It aims to assess *in vitro* antibacterial activity of *Lactobacillus* spp. strains against virulent *Salmonella* spp. isolated from slaughter animals in Benin.

## Materials and Methods

### Ethical approval

The manuscript does not contain clinical studies or patient data. The approval of the ethics committee was not required. However, samples were collected as per standard sample collection methods.

### Sampling

Samples of raw cow’s milk, breast milk, and infant feces were collected from cattle farms, nursing mothers, and healthy children (without sex distinction) aged 0-5 years, respectively. For breast milk and infant stool samples, informed consent of nursing mothers and one of the parents was obtained before sampling. All samples were aseptically made in sterile Stomacher bags and/or sterile pots. Each sample was identified and placed in a cooler containing cold accumulators. These samples were immediately sent to Research Unit in Applied Microbiology and Pharmacology of natural substances, University of Abomey-Calavi, for analysis. The objective was to evaluate the probiotic potential and anti-*Salmonella* activity of *Lactobacillus* strains isolated from cow’s milk samples. Hence, it was not necessary to evaluate the influence of external factors on the composition of the microbiome of cattle.

### Enrichment, isolation, and purification of Lactobacillus strains

Five grams or 5 ml of each sample were separately transferred aseptically into 45 ml of Man, Rogosa and Sharpe (MRS) broth (M1164-500G, HIMEDIA, India) and incubated for 24-48 h at 37°C for the enrichment of *Lactobacillus*.

To isolate the *Lactobacillus* strains, ten-fold serial dilution 10^−1^-10^−5^ was prepared from the pre-enriched broths. One milliliter of each dilution was inoculated on MRS agar w/low pH (M1927-500G, HIMEDIA, India) at the rate of three dishes by dilution. The Petri dishes were incubated anaerobically at 37°C for 24-72 h.

To purify the *Lactobacillus* strains, several successive subcultures were carried out on MRS agar. Transplanting and resuspension were performed only for very distinct, homogeneous, and well-developed colonies. The purity of the strain was verified by macroscopic and microscopic examination. Isolated *Lactobacillus* strains were stored on MRS broth with 40% (v/v) sterile glycerol (99%) at −20°C.

### Identification of isolates

The identification of species of *Lactobacillus* genus was based on the study of physiological characters (macroscopic and microscopic examination after Gram-staining, growth at different temperatures, and culture on hostile environments) and biochemical characters (catalase, indole, oxidase, mannitol mobility, Triple Sugar Iron [TSI] agar, and fermentation of sugars).

#### Physiological characters

Macroscopic and microscopic examination

The macroscopic examination consisted of describing the colonies obtained after culture on solid MRS medium of the bacterial strain during 48 h: According to pigmentation, contour, and aspect. After Gram-staining, microscopic examination allowed us to retain Gram-positive bacilli or coccobacilli.

Growth at different temperatures

A pure colony of each *Lactobacillus* isolate was emulsified into MRS broth and incubated at 15, 30, and 45°C for 48 h. The results (growth by turbidity) were read after 48 h for the last two temperatures and for 4 days for the test at 15°C. These tests allowed us to evaluate the ability of bacterial isolates to grow over a wide range of temperatures.

Culture on hostile environments

Adding 6.5 and 4%, NaCl (Cooper, French, 1304C028A) in MRS broth prepared the high salt medium. The culture on saline medium was studied by inoculation of one colony of each *Lactobacillus* isolate; the incubation was carried out at 37°C for 48 h in anaerobiosis, any positive reaction results in the presence of turbidity. Hundred microliters of these broths obtained after 48 h were inoculated on MRS agar. *Lactobacillus* colonies obtained on MRS agar plates after anaerobic incubation at 37°C for 24 h were counted.

#### Biochemical characters

The isolated *Lactobacillus* strains were subjected to catalase (using H_2_O_2_ on glass slide), oxidase (using oxidase discs), and indole test.

Study of fermental type and mobility test

Homofermentative and heterofermentative tests were performed in TSI agar medium. A pure and young *Lactobacillus* colony was collected and seeded by streaks on the slope and by a deep puncture in the TSI (Sterile Screw Tube) agar. The tubes thus seeded will be incubated at 37°C for 48 h under anaerobic conditions. The development of a homofermentary species does not cause discontinuity in the medium. Moreover, the presence of bubbles in the culture medium reflects a CO_2_ release characteristic of heterofermentary species. Apart from the fermental type, TSI agar has also allowed us to read the fermentation of lactose and glucose.

Mannitol is a product for the reduction of D-mannose. It allows us to simultaneously researching the fermentation of mannitol and mobility. The isolates studied were inoculated into the mannitol medium, and incubated at 37°C under anaerobic conditions for 48 h.

Fermentation of sugars

In addition to lactose, glucose, and mannitol, the fermentation of fructose, arabinose, maltose, galactose, and sucrose was studied. This sugar fermentation test was carried out on MRS plus phenol red medium free of meat extract and glucose with the addition of a pH indicator. We have tested the fermentation of various added sugars as the only carbon source in MRS red phenol.

The sugar solutions were prepared at 1% in sterilized distilled water by heating at 100°C for 30 min. From the young cultures of the strains, a culture colony was put in a sterile hemolysis tube with physiological saline, and then two successive centrifugations (3000 rpm for 20 min) were carried out to ensure a washing, in following 3 ml of MRS plus phenol red were added to the pellet. On a microplate 10 µl of different sugars were distributed in then, already containing 100 µl of the bacterial suspension. An MRS broth control was performed. Finally, the microplates were incubated at 37°C for 24-48 h. A positive result was a color shift [9].

### Assessment of in vitro probiotic abilities

#### Ability to survive in simulated gastric conditions

To study the probiotic properties of identified *Lactobacillus* strains tests for tolerance to pepsin (simulated gastric juice) and low-pH (acid), following the protocol of Huang and Adams [10]. Many studies have used acidity alone to evaluate the survival of probiotic strains during the passage of the stomach. In our study, it seemed interesting to test our strains against the effect of low pH alone and with respect to simulated gastric conditions (low pH effects with pepsin). Effect of pH 1.5, alone, on *Lactobacillus* spp. strains were examined after 1 h and 2 h exposure.

With respect to the assessment of the capacity of *Lactobacillus* spp. strains isolated to survive under conditions mimicking those of the human stomach, we prepared the simulated gastric juice by adding a solution of 0.5% NaCl (W/V) to a solution of pepsin concentration 3 g/l. The preparation was adjusted to pH 1.5. One milliliter of each bacterial culture was inoculated into 9 ml of the simulated gastric juice. Then, 100 µl of the seeded gastric juice was taken at 0 h and 2 h of exposure and inoculated by spreading on MRS agar. The number of colony-forming units (CFU) was determined after 48 h of anaerobic incubation. The experiment was repeated 3 times. The survival rate will be calculated by the following equation: Survival rate (%) = log CFU to T_2h_/log CFU to T_0h_×100.

#### Antibacterial activity

The antibacterial activity of *Lactobacillus* spp. strains isolated was determined by agar overlay method, against three strains of *Salmonella* spp., multidrug-resistant, and virulent isolated from slaughter animals in Southern Benin and one reference strain of *Salmonella* Typhimurium ATCC 14028 (Table-1) [11]. These strains were obtained at the Research Unit in Applied Microbiology and Pharmacology of Natural Substances of the University of Abomey-Calavi, Benin [11].

**Table-1:** Characteristics of *Salmonella* spp. strains (indicator strains) used [11].

Strains	Virulence genes				

	*invA*	*spvR*	*spvC*	*fimA*	*Stn*
P9	+	+		+	+
P14	+			+	+
P19	+	+	+	+	+
*Salmonella* Typhimurium ATCC 14028	+	+	+	+	+

P9; P14; P19; P29 are identified *Salmonella* spp. strains; +=Presence, *fimA*=Fimbriae production factor, *invA*=Invasion factor, *SpvR* and *SpvC*=Systemic infection (inhibition activation of macrophage), *Stn*=Enterotoxigenic substances production

The *Lactobacillus* strains isolated were spot inoculated, separately, onto the MRS agar plates, using a sub-culture (20 µl/spot) of MRS broth culture (grown at 37°C for 24 h) of the *Lactobacillus* strains, and the inoculated plates were incubated at 37°C for 24 h. The MRS agar plates containing the growth of *Lactobacillus* strains in spot form were thereafter overlaid with soft Mueller-Hinton agar (Biokar, French, BK048HA) pre-mixed with 100 µl (McFarland 1) of *Salmonella* spp. strains and *Salmonella* Typhimurium ATCC 14028 and incubated, after solidification of the overlaid agar medium, at 37°C for 24 h. The zone diameter of inhibition (ZDI) values obtained were measured and interpreted according to Halder *et al*. [12].

The “R” (width of clear zone) values were also determined as per the formula stated earlier [12]: R=(d Inhib–d Spot)/2; (d Inhib=the diameter of clear zone around the “d Spot;” and d Spot=the diameter of spot form of lactobacilli grown on MRS agar plate).

### Statistical analysis

All tests were repeated thrice, and the data were represented as mean±standard deviation.

## Results

### Physiological characters of isolates

**Figure-1:**
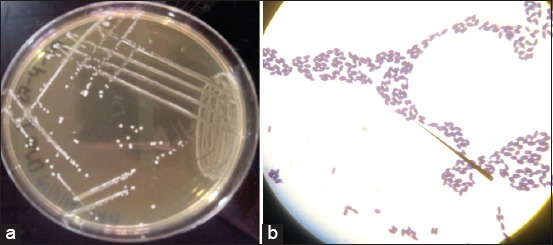
(a) typical colony characteristics of the isolates grown on MRS agar medium; (b) Microscopic view of the isolates when Gram stained.

**Figure-2:**
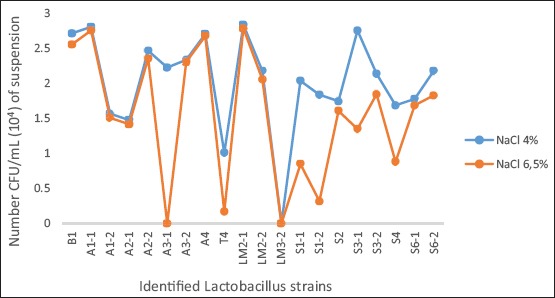
Tolerance of identified *Lactobacillus* strains with sodium chloride.

Of the 22 samples analyzed, 20 bacterial strains belonging to the genus *Lactobacillus* were isolated. The isolates were grown on selective MRS agar medium and gave a round or lenticular shape, whitish in color with regular outline (Figure-1a). After Gram-staining, we observed Gram-positive, short-medium, and sometimes diploid bacilli or coccobacilli (Figure-1b). The majority of isolates were able to grow in MRS broth at different temperatures except at the temperature of 15°C. The cultivation of the strains in the presence of different concentrations of NaCl 6.5 and 4%, allowed us to evaluate their ability to grow in hostile conditions. The majority of identified strains have resisted the different concentrations of NaCl solution to which they are subjected. Nevertheless, *Lactobacillus casei* A3-1, *Lactobacillus plantarum* LM3-2, and *Lactobacillus acidophilus* T4 strains were unable to resist the 6.5% NaCl solution (Figure-2).

### Biochemical characterization

Catalase, oxidase, and indole tests performed as part of the biochemical characterization of the isolates gave negative results for all isolates. The distribution according to fermental type was unequal between strains. The homofermentative strains represent almost all the number of isolates. The determination of genera and bacterial species lies mainly in their ability to ferment sugars lactic acid and other organic acids. The analysis of the fermental profiles reveals a great metabolic diversity of the carbohydrates in the isolates. Carbohydrate utilization test was performed to investigate whether the isolates can ferment lactose, sucrose, glucose, maltose, galactose, fructose, arabinose, and mannitol (Table-2). Of the 20 strains identified, nine (45%) were isolated from raw cow milk samples with a high proportion of *L. casei* (55.56%) followed by *L. plantarum* (33.33%). About 40% (8/20) of the strains identified came from infantile stool samples, with 50% *Lactobacillus delbrueckii* and 37.50% *Lactobacillus salivarius*. About 15% (3/20) of the strains identified came from breast milk samples with a high proportion of *L. plantarum* (66.67%). After identification of the strains, the most represented species is *L. plantarum* (30%), followed by *L. delbrueckii* (25%), *L. casei* (25%), *L. salivarius* (15%), and *L. acidophilus* (05%) (Table-3).

**Table-2:** Results of the biochemical tests carried out on isolates.

Identified species (Isolates code)	Parameters													

	Catalase	Oxidase	Indole	Growth at 45°C	Sugar fermentation									

					Ara	Lac	Glu	Fru	Gal	Man	Mal	Suc	Raf	Rha
*Lactobacillus salivarius (S6-1; S6-2; S3-1)*	-	-	-	+	-	+	+*	+	+	+	+	+	+	+
*Lactobacillus acidophilus (T4)*	-	-	-	+	-	+	+*	+	+	-	+	+	-	-
*Lactobacillus delbruekii (S3-2; S2; S1-2; S1-1; LM2-1)*	-	-	-	+	-	+	+*	+	+	-	+	+	+	-
*Lactobacillus casei (A1-2; A2-2; A3-1; A4; B1)*	-	-	-	+	-	+	+*	+	+	+	+	+	-	-
*Lactobacillus plantarum (S4; LM2-2; A3-2; A1-1; A2-1; LM3-2)*	-	-	-	+	+	+	+*	+	+	+	+	+	+	+

Ara: Arabinose; Lac: Lactose; Glu: Glucose; Fru: Fructose; Gal: Galactose; Man: Mannitol; Mal: Maltose; Suc: Sucrose; Raf: Raffinose; Rha: Rhamnose; *No gas production from glucose

**Table-3:** Distribution of identified *Lactobacillus* strains according to different types of samples.

Sample types	Identified bacterial species					Total

	*L. salivarius*	*L. acidophilus*	*L. delbrueckii*	*L. casei*	*L. plantarum*	
Infantile stool	3 (37.50%)	-	4 (50%)	-	1 (12.50%)	8
Raw cow milk	-	1 (11.11%)	-	5 (55.56%)	3 (33.33%)	9
Breast milk	-	-	1 (33.33%)	-	2 (66.67%)	3
Total	3 (15%)	1 (05%)	5 (25%)	5 (25%)	6 (30%)	20

### Probiotic activity

#### Ability of Lactobacillus spp. isolates to survive in gastric conditions

**Figure-3:**
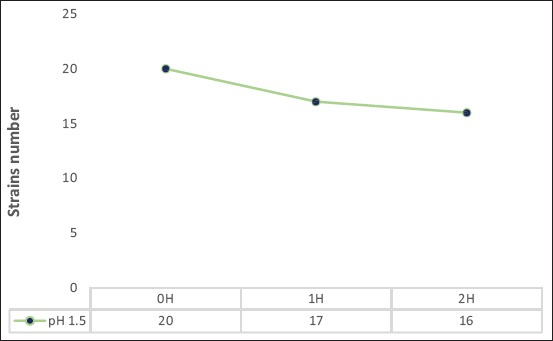
Effect of pH 1.5 on identified *Lactobacillus*.

To exercise their probiotic power, the strains must be able to survive the gastric conditions *in vivo*. This is how the present study tested their survival in simulated gastric conditions *in vitro*. From Figure-3, it appears that pH 1.5, alone, has a drastic effect on some of the identified strains. In fact, of the 20 *Lactobacillus* strains tested, 17 resisted after 1 h of incubation, and 16 showed remarkable resistance after 2 h of exposure. It thus appears that the sensitivity of *Lactobacillus* to the acidic pH depends on the strain since even the acid-fast strains have shown, differently, more or less interesting survival rates, ranging from 20% to 83%, 50% (Table-4). Of the 16 strains resistant to pH 1.5, only 14 resisted stimulated gastric conditions (pH 1.5+pepsin). The strains of *L. salivarius* S6-1 and S6-2 were completely inhibited. The highest resistance rate was observed with *L. salivarius* S3-1 (60.76%), followed by *L. casei* B1 (60%), *L. plantarum* S4 (57.55%), *L. delbrueckii* S2 (53%), and *L. delbrueckii* S3-2 (52.58%) (Table-4).

**Table-4:** Ability of lactobacilli strains to survive in simulated gastric conditions.

Identified Lactobacillus strains	Codes	Viable cultures number (log cfu.mL^−1^)* at times 0H and 2H			

		Simulated gastric juice	OH	2H	Survival rate (%)
*L. salivarius*	*S3-1*	pH 1.5	2.37±0.46	1.81±0.01	73.37
		pH 1.5+pepsin		1.44±0.02	60.76
	*S6-1*	pH 1.5	1.85±0.02	0.42±0.03	22.70
		pH 1.5+pepsin		0	0
	*S6-2*	pH 1.5	1.40±0.01	0.28±0.02	20
		pH 1.5+pepsin		0	0
*L. delbrueckii*	*S1-1*	pH 1.5	1.82±0.02	1.22±0.03	63.03
		pH 1.5+pepsin		0.88±0.02	48.35
	*S2*	pH 1.5	2.34±0.03	1.81±0.02	77.35
		pH 1.5 +pepsin		1.24±0.02	53
	*S3-2*	pH 1.5	1.94±0.04	1.620.01	83.50
		pH 1.5+pepsin		1.02±0.04	52.58
	*LM2-1*	pH 1.5	2.45±0.01	0.90±0.02	36.73
		pH 1.5+pepsin		0.83±0.04	33.88
*L. casei*	*A1-2*	pH 1.5	2.31±0.02	1.30±0.02	56.28
		pH 1.5+pepsin		0.95±0.02	41.13
	*A2-2*	pH 1.5	2.36±0.01	1.65±0.01	69.92
		pH 1.5+pepsin		0.97±0.01	41.10
	*A4*	pH 1.5	2.82±0.03	0.98±0.02	34.75
		pH 1.5+pepsin		0.62±0.03	21.99
	*B1*	pH 1.5	2.30±0.01	1.73±0.02	75.22
		pH 1.5+pepsin		1.38±0.07	60
*L. plantarum*	*A1-1*	pH 1.5	2.48±0.01	1.52±0.04	61.29
		pH 1.5+pepsin		1.05±0.02	42.34
	*A2-1*	pH 1.5	2.95±0.03	1.77±0.02	60
		pH 1.5+pepsin		1.19±0.01	40.34
	*A3-2*	pH 1.5	2.31±0.01	1.70±0.02	73.59
		pH 1.5+pepsin		0.99±0.02	42.86
	S4	pH 1.5	2.12±0.02	1.70±0.01	80.19
		pH 1.5+pepsin		1.22±0.03	57.55
	LM2-2	pH 1.5	1.71±0.01	0.77±0.03	45.03
		pH 1.5+pepsin		0.42±0.03	24.56

(log cfu.mL-1)*: the values of log CFU.mL.^-1^ express mean±standard deviation, each data point is mean of repeated measurements of 3 experiments independent, n=3. p<0.05.

#### Anti-Salmonella spp. activity of Lactobacillus spp. isolates

**Figure-4:**
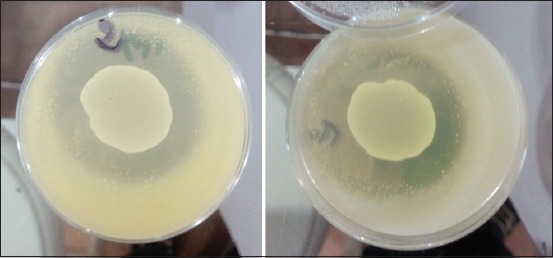
Antimicrobial Activity of isolated *Lactobacillus* spp. Clear zone indicates inhibition of bacterial growth.

The identified *Lactobacillus* strains showed excellent antibacterial activity against *Salmonella* spp. strains tested (Figure-4). The *L. salivarius* S3-1 strains had top growth inhibitory activity against three virulent and multidrug-resistant *Salmonella* spp. used and *Salmonella* Typhimurium ATCC 14028 with ZDI ranging from 30.75±0.06 mm to 35.33±0.05 mm. The *L. plantarum* A1-1 and LM2-2 had poor antibacterial activity against indicator strains used with ZDI ranging from 12.36±0.03 mm to 16.25±0.23 mm (Table-5).

**Table-5:** Antibacterial activity of the identified Lactobacillus strains, in terms of ZDI.

Identified Lactobacillus strains	Codes	ZDI (mm), Mean±SD for indicator strains			

		P9	P14	P19	*Salmonella Typhimurium* ATCC 14028
*L. salivarius*	*S3-1*	35.33±0.05	33.37±0.46	30.81±0.01	34.23±0.52
*L. delbrueckii*	*S1-1*	31.52±0.24	30.82±0.02	28.22±0.03	29.42±0.35
	*S2*	29.25±0.45	27.34±0.03	27.31±0.02	25.84±0.64
	*S3-2*	23.62±0.35	21.94±0.04	21.62±0.01	22.41±0.01
	*LM2-1*	26.16±0.02	26.45±0.01	23.90±0.02	25.13±0.54
*L. casei*	*A1-2*	18.56±1.05	17.31±0.02	16.3±01.25	16.38±0.52
	*A2-2*	28.50±0.01	24.36±0.01	27.65±00.01	26.25±0.24
	*A4*	33.43±0.54	32.82±0.03	34.98±00.02	34.28±0.04
	*B1*	33.84±0.63	34.30±0.01	31.38±00.07	30.75±0.06
*L. plantarum*	*A1-1*	14.65±0.58	12.48±0.01	13.52±00.04	12.36±0.03
	*A2-1*	24.65±0.35	21.95±0.03	21.77±0.02	20.64±0.03
	*A3-2*	31.25±0.02	30.31±0.01	30.70±00.02	31.54±0.04
	S4	28.65±0.56	26.12±0.02	26.70±00.01	26.84±0.05
	LM2-2	16.25±0.23	15.71±0.01	15.77±00.03	14.95±0.02

The “R” values of identified *Lactobacillus* strains against *Salmonella* spp. strains used are represented in Table-6. *L. plantarum* A1-1 and LM2-2 have very low inhibition against *Salmonella* spp. strains and against *Salmonella* Typhimurium ATCC 14028 with R-values between 02.45±0.43 mm and 06.30±0.45. On the other hand, all the other strains of *Lactobacillus* identified have strong inhibition against the same strains (R>6 mm).

**Table-6:** The « R » values of identified Lactobacillus strains against the indicator strains used.

Identified Lactobacillus strains	Codes	R Value (mm), Mean±SD, for indicator strains			

		P9	P14	P19	*Salmonella Typhimurium* ATCC 14028
*L. salivarius*	*S3-1*	16.05±0.29	15.37±0.35	13.75±0.51	15.67±0.41
*L. delbrueckii*	*S1-1*	14.33±0.29	13.62±0.01	13.08±0.23	13.46±0.29
	*S2*	13.35±0.35	12.67±0.65	12.41±0.01	10.75±0.46
	*S3-2*	10.23±0.35	09.54±0.02	05.14±0.21	10.04±0.31
	*LM2-1*	11.05±0.22	11.35±0.02	10.36±0.52	10.85±0.51
*L. casei*	*A1-2*	08.64±0.05	07.25±0.62	06.45±1.25	06.68±0.54
	*A2-2*	13.10±0.01	10.50±0.01	12.65±0.02	11.25±0.34
	*A4*	15.33±0.51	14.62±0.01	15.84±0.25	15.68±0.34
	*B1*	15.54±0.02	15.50±0.03	14.08±0.05	13.49±0.51
*L. plantarum*	*A1-1*	04.52±0.48	02.54±0.06	03.52±0.25	02.45±0.43
	*A2-1*	10.50±0.45	09.85±0.01	09.67±0.01	09.15±0.53
	*A3-2*	14.35±0.52	13.65±0.51	13.72±0.32	14.25±0.45
	S4	13.10±0.51	11.25±0.42	11.85±0.21	11.94±0.04
	LM2-2	06.30±0.45	05.61±0.04	05.87±0.05	04.79±0.42

## Discussion

The aim of this study was to evaluate the probiotic potential of some strains of *Lactobacillus* spp. to inhibit the *in vitro* the growth of virulent and multi-resistant strains of *Salmonella* spp. isolated from slaughter animals. To do this, it was a question of identifying strains of *Lactobacillus* spp. probiotic from breast milk, cow’s milk, and infant stool samples and to assess their anti-*Salmonella* spp. activity. Isolation and purification of lactobacilli on the MRS agar medium led to the collection of 20 strains. Since lactobacilli are bacilli, microscopic examination of the isolates allowed us to observe 75% of bacilli and 25% of all Gram-positive coccobacilli. The results of the microscopic, macroscopic, and above all biochemical examination (fermentation of sugars) made it possible to identify several species, such as *L. plantarum* (30%), *L. delbrueckii* (25%), *L. casei* (25%), *L. salivarius* (15%), and *L. acidophilus* (05%).

Probiotic strains, to be effective, must arrive alive to the site of their action, namely the intestine and therefore resist during their passage to the hostile conditions of the stomach such as acidity and the action of pepsin [13]. While sodium chloride inhibits the growth of many other types of bacteria, probiotic bacteria resist high levels of salt in the human gut [14]. The majority of *Lactobacillus* isolates were resistant to the various NaCl solution (4-6.5%) except *L. casei* A3-1 and *L. plantarum* LM3-2, which could not withstand NaCl 6.5% solution. All *L. casei* strains are isolated from cow milk samples (A1-2; A2-2; A3-1; A4; and B1), apart from the A3-1 strain, the others are resistant to NaCl (4-6.5%). These results are similar to those of Halder and Mandal [14] who showed that all *L. casei* strains isolated from curd samples are resistant to NaCl solution (4-6.5%).

During fasting, the stomach pH can go down very low, up to 1.5 in some people, which can fatally affect bacterial growth [15]. In our study, *Lactobacillus* isolates showed very different behaviors with respect to their sensitivity to pH 1.5. This had a drastic effect on four identified strains of *Lactobacillus* (*L. acidophilus* T4, *L. delbrueckii* S1-2, *L. casei* A3-1, and *L. plantarum* LM3-2) but 16 strains resisted this strong acidity after 2 h of incubation. It was demonstrated that for probiotic characterization of lactobacilli, acid tolerance is an important criterion, and the pH value of 3.0 has been considered standard for such investigation of probiotic strains [3]. Thus, the 16 strains resistant to pH 1.5 are potential probiotic strains. However, it can be seen that pH alone is not a good criterion for evaluating the resistance of probiotic strains during their passage through the stomach. Two of the 16 strains resistant to pH 1.5 could not withstand the simulated gastric conditions (pH 1.5+pepsin). These are *L. salivarius* strains S6-1 and S6-2. Strains resistant to simulated gastric juice: *L. salivarius* S3-1, *L. delbrueckii* (S1-1, S2, S3-2, and LM2-1), *L. casei* (A1-2, A2-2, A4, and B1), and *L. plantarum* (A1-1, A2-1, A3-2, S4, and LM2-2) showed more or less interesting and variable survival rates from one strain to another. With regard to the best survival rate of isolates under-stimulated gastric conditions, *L. delbrueckii* S3-2 and *L. plantarum* S4 lead with, respectively, 83.50% and 80.19% (Table-4). These results are similar to those obtained by Sirichokchatchawan *et al*. [16], which showed that *L. plantarum* isolated from pig feces was higher tolerance to stimulated gastric conditions with a survival rate of 90.71%.

Antagonist activity is one of the functional requirements of probiotics. In this study, virulent and multi-resistant *Salmonella* spp. strains were selected as indicator bacteria. The *Lactobacillus* strains studied showed different anti-*Salmonella* spp. activities. The most active strains are *L. salivarius* S3-1, *L. delbrueckii* S1-1, *L. casei* A4 and B1, and *L. plantarum* A3-2 with ZDIs of 28.22±0.03 mm-35.33±0.05 mm (Table-5). These results are similar to those obtained by Sirichokchatchawan *et al*. [16], where strains of *L. plantarum* isolated from pig feces have a strong antibacterial activity on *Salmonella* Typhimurium ATCC 13311 and *Salmonella choleraesuis* with ZDIs >17 mm. Likewise, our results are similar to those of Adetoye *et al*. [3] and Halder *et al*. [12]. Adetoye *et al*. [3] showed that two *L. plantarum* strains and one *L. salivarius* strain isolated from cattle feces had a strong antibacterial activity on *Salmonella enterica* strains with, respectively, ZDIs of 12-18 and >18 mm. As for Halder *et al*. [12], they showed that *L. plantarum* strain isolated from curd samples had a strong antibacterial activity on *Salmonella* Typhi of human origin (ZDI=25.25±1.71 mm). Our results are better than those obtained by Yamazaki *et al*. [17], where strains of *L. delbrueckii*, *L. plantarum*, *L. casei*, and *L. salivarius* isolated from chicken feces had low anti-Salmonella spp. activity with ZDIs ranging from 1.3±0.4 mm to 11.7±1.0 mm. According to Halder *et al*. [12], when “R” was <2 mm that the scores of growth inhibition of indicator bacteria were considered as no inhibition capacity; low inhibition capacity with “R” values of 2-5 mm, and high inhibition capacity with “R” values >6 mm. It should be noted that all selected strains of *Lactobacillus* have a strong inhibition (R>6 mm) on virulent strains of *Salmonella* spp.

## Conclusion

*Lactobacillus* strains isolated from cow milk, breast milk, and infantile stool might be used as valid candidates of probiotics and good alternatives for antibiotics in the fight against animal and human salmonellosis. To the best of our understanding, this is the first study unfolding the antagonistic activity of 14 *Lactobacillus* strains against pathogenic bacteria in Benin, and the probiotic potential of such lactic acid bacteria has been authenticated. However, further studies are required to explore the effectiveness of the antibacterial essence of such native lactobacilli, to be used as the alternative therapeutics, in combating bacterial antibiotic resistance and treating the infections.

## Authors’ Contributions

AJA and TVD wrote the protocol. AJA, TVD, RB, and ED processed the samples and laboratory analysis. AJA did data processing and statistical analysis. AJA and RB wrote the manuscript. TVD and LB reviewed the manuscript. All authors read and approved the final manuscript.

## References

[1] Abhisingha M, Dumnil J, Pitaksutheepong C (2018). Selection of potential probiotic *Lactobacillus* with inhibitory activity against *Salmonella* and fecal coliform bacteria. Probiotics Antimicrob. Proteins.

[2] Hung Y.T, Lay C.J, Wang C.L, Koo M (2017). Characteristics of non-typhoidal gastroenteritis in Taiwanese children:A 9year period retrospective medical record review. J. Infect. Public Health.

[3] Adetoye A, Pinloche E, Adeniyi B.A, Ayeni F.A (2018). Characterization and anti-salmonella activities of lactic acid bacteria isolated from cattle feces. BMC Microbiol.

[4] Dougnon T.V, Deguenon E, Fah L, Legba B, Hounmanou Y.M, Agbankpe A.J, Amadou A, Koudokpon H, Fabiyi K, Aniambossou A, Assogba P, Hounsa E, de Souza M, Avlessi F, Dougnon T.J, Gbaguidi F, Boko M, Bankolé H.S, Baba-Moussa L (2017). Traditional treatment of human and animal salmonellosis in Southern Benin:Knowledge of farmers and traditherapists. Vet. World.

[5] Das J.K, Mishra D, Ray P, Tripathy P, Beuria T.K, Singh N, Suar M (2013). *In vitro* evaluation of anti-infective activity of a *Lactobacillus plantarum* strain against *Salmonella enterica* serovar Enteritidis. Gut Pathog.

[6] Dibong S.D, Mpondo E.M, Ngoye A, Kwin M.F, Betti J.L (2011). Ethnobotanique et phytomédecine des plantes médicinales de Douala. J. Appl. Biosci.

[7] Potočnjak M, Pušić P, Frece J, Abram M, Janković T, Gobin I (2017). Three new *Lactobacillus plantarum* strains in the probiotic toolbox against gut pathogen *Salmonella enterica* serotype Typhimurium. Food Technol. Biotechnol.

[8] Liu J, Gu Z, Song F, Zhang H, Zhao J, Chen W (2019). *Lactobacillus plantarum*ZS2058 and *Lactobacillus rhamnosus*GG use different mechanisms to prevent *Salmonella* infection *in vivo*. Front. Microbiol.

[9] Stiles M.E, Holzapfel W.H (1997). Review article lactic acid bacteria of foods and current taxonomy. Int. J. Microbiol.

[10] Huang Y, Adams M.C (2004). *In vitro* assessment of the upper gastrointestinal tolerance of potential probiotic dairy propionibacteria. Int. J. Food Microbiol.

[11] Deguenon E, Dougnon V, Lozes E, Maman N, Agbankpe J, Abdel-Massih R, Djegui F, Baba-Moussa L, Dougnon J (2019). Resistance and virulence determinants of fecal *Salmonella* spp isolated from slaughter animals in Benin. BMC Res. Notes.

[12] Halder D, Mandal M, Sekhar S, Kumar N, Mandal S (2017). Indigenous probiotic *Lactobacillus* isolates presenting antibiotic like activity against human pathogenic bacteria. Biomedicines.

[13] Dunne C, O'Mahony L, Murphy L, Thornton G, Morrissey D, O'Halloran S, Feeney M, Flynn S, Fitzgerald G, Daly C, Kiely B, O'Sullivan G.C, Shanahan F, Collins J.K (2001). *In vitro* selection criteria for probiotic bacteria of human origin:Correlation with *in vivo* findings. Am. J. Clin. Nutr.

[14] Halder D, Mandal S (2015). Curd lactobacilli with probiotic potentiality. Transl. Biomed.

[15] Draser B.S, Shiner M, McLeod G.M (1969). Studies on the intestinal flora I. The bacterial flora of the gastrointestinal tract in healthy and achlorhydric persons. Gastroenterology.

[16] Sirichokchatchawan W, Pupa P, Praechansri P, Am-In N, Tanasupawat S, Sonthayanon P, Prapasarakul N (2018). Autochthonous lactic acid bacteria isolated from pig feces in Thailand show probiotic properties and antibacterial activity against enteric pathogenic bacteria. Microb. Pathog.

[17] Yamazaki M, Ohtsu H, Yakabe Y, Kishima M, Abe H (2012). *In vitro* screening of lactobacilli isolated from chicken excreta to control *Salmonella* Enteritidis and Typhimurium. Br. Poult. Sci.

